# Label-free volumetric optical imaging of intact murine brains

**DOI:** 10.1038/srep46306

**Published:** 2017-04-12

**Authors:** Jian Ren, Heejin Choi, Kwanghun Chung, Brett E. Bouma

**Affiliations:** 1 Wellman Center for Photomedicine, Massachusetts General Hospital, Boston, MA 02114, USA; 2 Harvard Medical School, Boston, MA 02115, USA; 3 Institute for Medical Engineering and Science, Massachusetts Institute of Technology, Cambridge, MA 02139, USA; 4 Picower Institute for Learning and Memory, Massachusetts Institute of Technology, Cambridge, MA 02139, USA; 5 Department of Brain and Cognitive Sciences, Massachusetts Institute of Technology, Cambridge, MA 02139, USA; 6 Department of Chemical Engineering, Massachusetts Institute of Technology, Cambridge, MA 02139, USA; 7 Broad Institute of Harvard University and Massachusetts Institute of Technology, Cambridge, MA 02142, USA

## Abstract

A central effort of today’s neuroscience is to study the brain’s ’wiring diagram’. The nervous system is believed to be a network of neurons interacting with each other through synaptic connection between axons and dendrites, therefore the neuronal connectivity map not only depicts the underlying anatomy, but also has important behavioral implications. Different approaches have been utilized to decipher neuronal circuits, including electron microscopy (EM) and light microscopy (LM). However, these approaches typically demand extensive sectioning and reconstruction for a brain sample. Recently, tissue clearing methods have enabled the investigation of a fully assembled biological system with greatly improved light penetration. Yet, most of these implementations, still require either genetic or exogenous contrast labeling for light microscopy. Here we demonstrate a high-speed approach, termed as Clearing Assisted Scattering Tomography (CAST), where intact brains can be imaged at optical resolution without labeling by leveraging tissue clearing and the scattering contrast of optical frequency domain imaging (OFDI).

Clusters of neurons with diverse structural and functional traits comprise an astonishingly intricate network with interconnections spanning across the entire brain at both local and global scales. This network reflects not only the topological links among neurons but also their aggregate behavior leading to cognitive functions^1^. The ‘Connectome’, or brain-wide connectivity map^2^, lays the foundation for understanding how information is transmitted and processed in this extraordinary system.

In spite of its significance, mapping the comprehensive wiring diagram across the whole brain has been a daunting challenge for many years and our understanding on neuronal connectivity still remains staggeringly fragmented^3^. Toward this challenge, over the past decade numerous efforts have been made at multiple scales with various spatial resolution^4^,^5^,^6^. At the macroscale, magnetic resonance imaging (MRI) based modalities with a resolution from 1 *mm* to 100 *μm* are prevailing^4^. While functional MRI recognizes different brain regions based on their functions, diffusion-weighted MRI (dMRI) can infer structural connectivity by depicting white matter tracts connecting those regions. Although this method has been used to study cognitive and affective processes *in vivo*, the current resolution of one-cubic-millimeter makes wiring diagrams obtained through dMRI too coarse to disclose the complete neuronal network explicitly. At the opposite extreme, scanning electron microscopy (SEM) has a resolution of around 30 *nm* when applied to ultra thin, serially-sectioned specimens and is able to visualize the finest structure of neurons, such as synaptic connection^7^. At present, this microscale approach, owing to the extensive tissue preparation and the required large scale morphological reconstruction, is only feasible for investigations on small tissue volume^5^. To bridge these two scales, light microscopy (LM) provides a mesoscale alternative, offering a typical resolution ranging from a few hundred nanonmeters to 1~2 *μm *6. Being able to reveal both long-range and local connections, LM has facilitated the successful acquisition of various mesoscale connectomes of the mouse brain^8^,^9^,^10^. Light scattering in tissue, however, poses a stringent limit on the imaging depth of optical methods. While sectioning samples into a sequence of thin slices is a solution to this limited imaging depth^8^,^10^, it still suffers from tissue deformation caused by the mechanical sectioning and therefore the reconstruction of 3D volume from 2D images can be challenging^6^,^11^.

Another approach for deep penetration optical imaging without sectioning, tissue clearing, originated decades ago^12^ has recently received renewed interest^13^. Through refractive index matching by either organic^14^,^15^,^16^,^17^ or aqueous solvents^18^,^19^,^20^,^21^ and with additional lipid removal from cell membranes^15^,^16^,^20^,^21^,^22^,^23^, imaging through thick tissues or even the whole murine brain have been achieved. Tissue clearing indeed provides a powerful tool to investigate intact biological systems. Although some attempts were made to image mouse brains without labeling^24^,^25^, the insufficient light penetration prevents them from whole-brain imaging. As a result, most existing LM methods for whole-brain imaging, whether with tissue clearing or without, still rely on either genetic or exogenous reporters to provide contrast. While immunostaining and reporter genes do offer great molecular and cellular specificity, they all require substantial sample preparation. First, genetic contrast is largely limited to the transgenic models or viral labeling current available. It is also not compatible with some clearing methods due to fluorescence quenching during the clearing process^14^,^15^,^16^. Immunohistochemistry provides a wide spectrum of biomarkers for cell phenotyping, but staining large samples homogeneously remains very challenging due to enormous molecular barriers, such as lipid bilayer and protein cross-link inside the sample volume^22^,^23^. Thus, despite recent efforts aiming to facilitate this process^26^,^27^, fully cleared and uniformly labeled samples are not yet easily accessible to researchers.

To address this, here we introduce a high-speed label-free methodology, termed as Clearing Assisted Scattering Tomography (CAST), for imaging intact brains. Rather than sensing the incoherent photons emitted by fluorophores or absorbed by chromophores, optical frequency domain imaging (OFDI) and optical coherence tomography (OCT) coherently detect photons scattered back from tissue^28^. Based solely on intrinsic tissue scattering, OFDI allows for high-speed imaging without the need for labeling, which is remarkably advantageous for high throughput investigation on intact tissues. While deeper than confocal (tens of micron)^29^ and two-photon microscopy (hundreds of micron)^30^, all existing OFDI/OCT imaging are still limited to 1~2 millimeters by tissue scattering. This prevents all previous OFDI/OCT studies^31^,^32^,^33^ from imaging deep into an intact brain. As we will show in this work, by adapting the OFDI apparatus and controlling photon scattering through the clearing procedure, CAST enables high resolution, cross-sectional and volumetric imaging through thick intact tissue without any contrast agent. The significant merits of this new method CAST, lie in that it provides a unique way to image deep tissue at optical resolution in a label-free manner.

## Results

**Figure 1:**
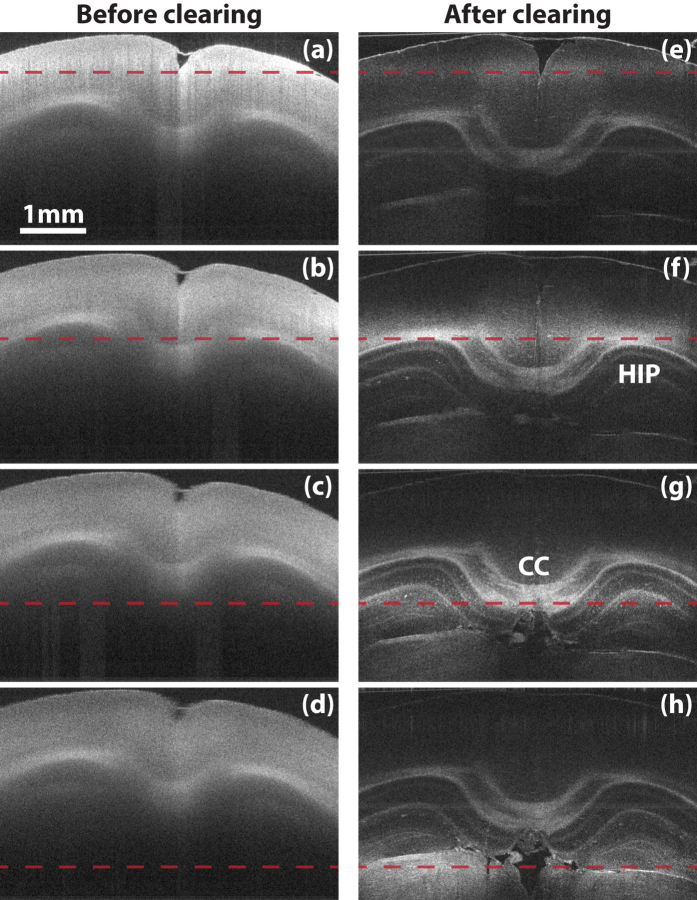
OFDI penetration enhancement. (**a**–**d**) The coronal images of a fixed mouse brain while moving the optical focus deep by a step of 707 *μm*. (**e**–**h**) The coronal images of the same section after clearing with the same location of focal plane, indicated by the dashed lines. All images share the same scale bar in (**a**). CC: Corpus callosum; HIP: Hippocampus.

While clearing indeed extends light penetration in tissue, it inevitably decreases OFDI signal due to scattering reduction. By adjusting the degree of lipid removal, we found that it was possible for CAST to image through very thick tissue while maintaining sufficient contrast. To introduce OFDI imaging on cleared samples, we first constructed an OFDI imaging system (Method). This platform was designed to have a nearly isotropic optical resolution volume of 10 × 10 × 9 *μm*^3^ (*X* × *Y* × *Z*) in air to demonstrate that CAST can work efficiently at the *resolution gap* between conventional LM (1 *μm*) and MRI (100 *μm*). This configuration also yielded the best balance between sufficient structural visualization and scanning time. As will be shown later, a mouse brain hemisphere can be scanned in around 22 minutes. Figure 1(a–d) show OFDI cross-sections of a mouse brain with the optical focus at different depths. In comparison, Fig. 1(e–h) show cross-sections of the same sample after a controlled clearing (Method), with the foci at the corresponding locations as in Fig. 1(a–d). By moving the focus deeper into the tissue, anatomical structures at different depths emerged in the images of the cleared sample, while the regions that can be visualized in the images of the sample before clearing all remain only on the shallow cortical layers, regardless of the location of the focus. Although the imaging region in the cleared sample may be limited by the depth of focus of the optics, this result indicates that not only many photons reached deep structures of the brain but also a sufficient number of scattered photons were collected from deep in the brain to form the CAST tomogram.

**Figure 2:**
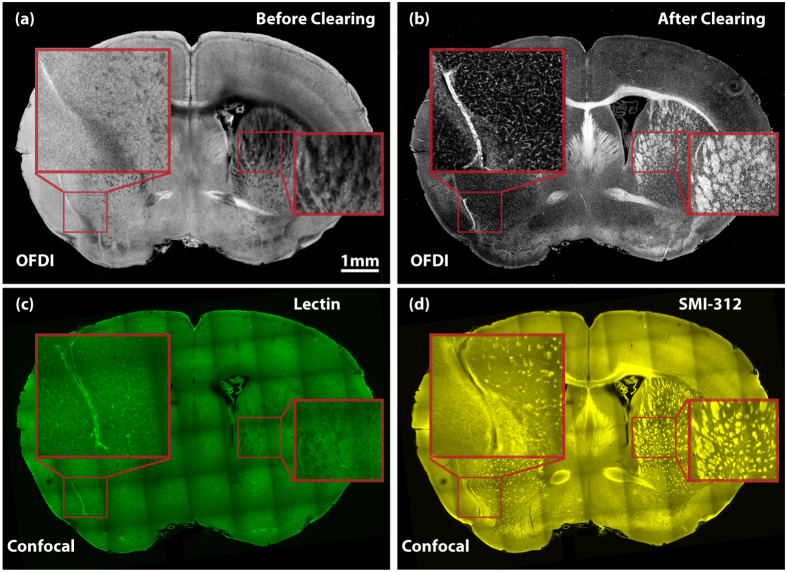
Contrast validation. (**a**) The OFDI image of a mouse brain coronal section before clearing. (**b**) The OFDI image of the same section in (**a**) after clearing. (**c**) The confocal image of the same section stained by lectin. (**d**) The confocal image of the same section stained by SMI-312. All images share the same scale bar in (**a**).

Next, we further investigated which biological structures within the cleared samples gave rise to OFDI signal. A 1 *mm* thick coronal section from a mouse brain was used to examine the biological origins of OFDI contrast. To discover how the clearing process affects the scattering signal, we first compared the OFDI images of the same coronal section before and after a controlled clearing process. As shown in Fig. 2(a and b), the signal strength of different parts were altered drastically during the clearing. To further validate OFDI contrast, the same tissue was stained with lectin (Fig. 2(c)) and SMI-312 (Fig. 2(d)), and then imaged using a confocal fluorescence microscope (Method). Both the neuronal fibers and the blood vessel labeled in the fluorescence images (SMI-312 and lectin) were strongly correlated to the structures with positive contrast in CAST images. Another important observation is that after clearing, the contrast of the neuronal bundles and blood vessel was generally enhanced across the entire section while scattering from most of other tissue components was suppressed. This demonstrates that clearing can indeed selectively enhance the contrast of some important anatomical structures in mammalian brains.

**Figure 3:**
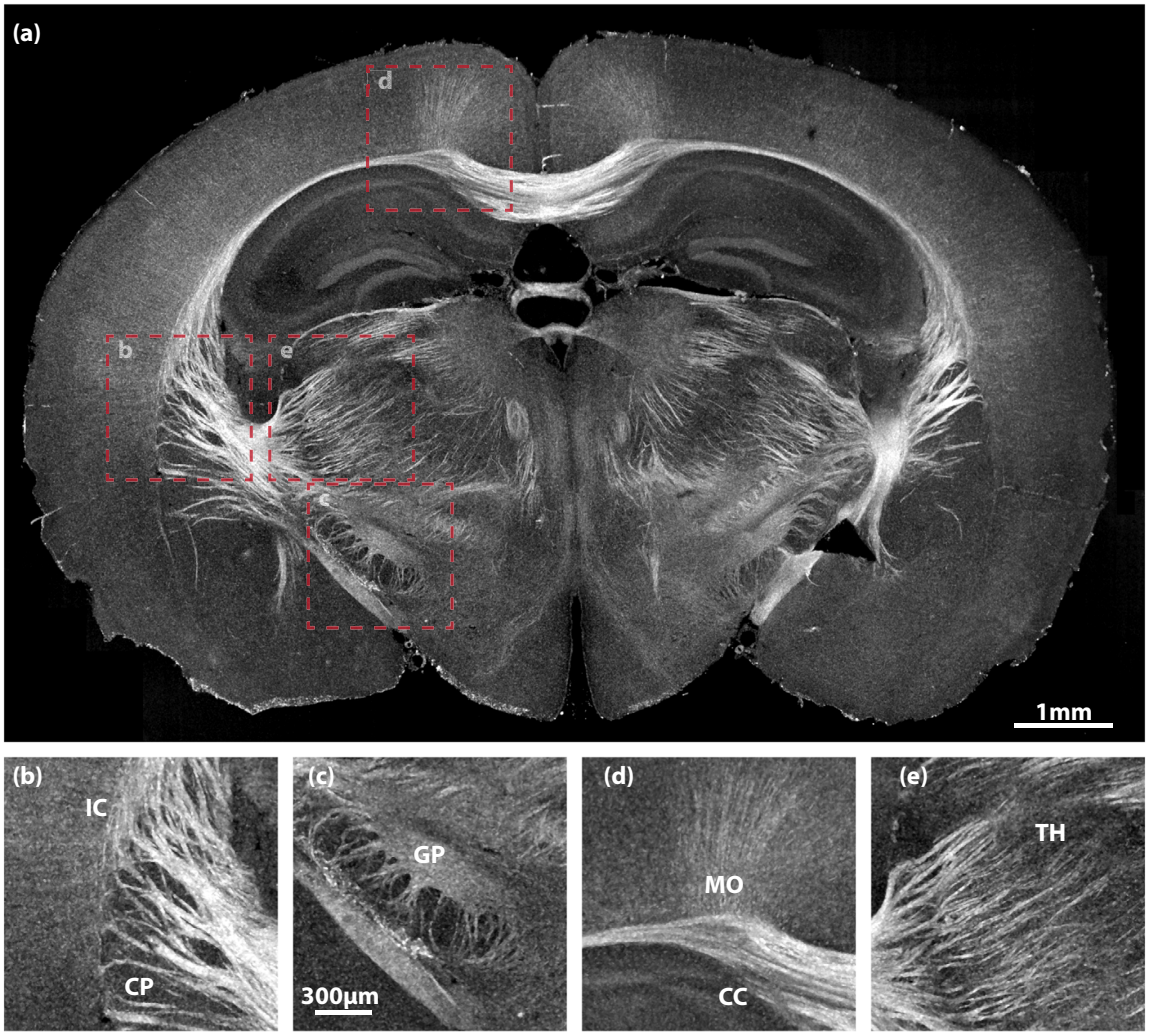
CAST imaging of a 1 *mm* thick mouse brain section. (**a**) A coronal view of the section. (**b**–**e**) Magnified images of the boxed areas in (**a**). (**b**) Caudoputamen (CP) and internal capsule (IC); (**c**) Globus pallidus (GP); (**d**) Corpus callosum (CC) and cortical motor area (MO); (**e**) Thalamus (TH). (**b**–**e**) share the same scale bar in (**c**).

**Figure 4:**
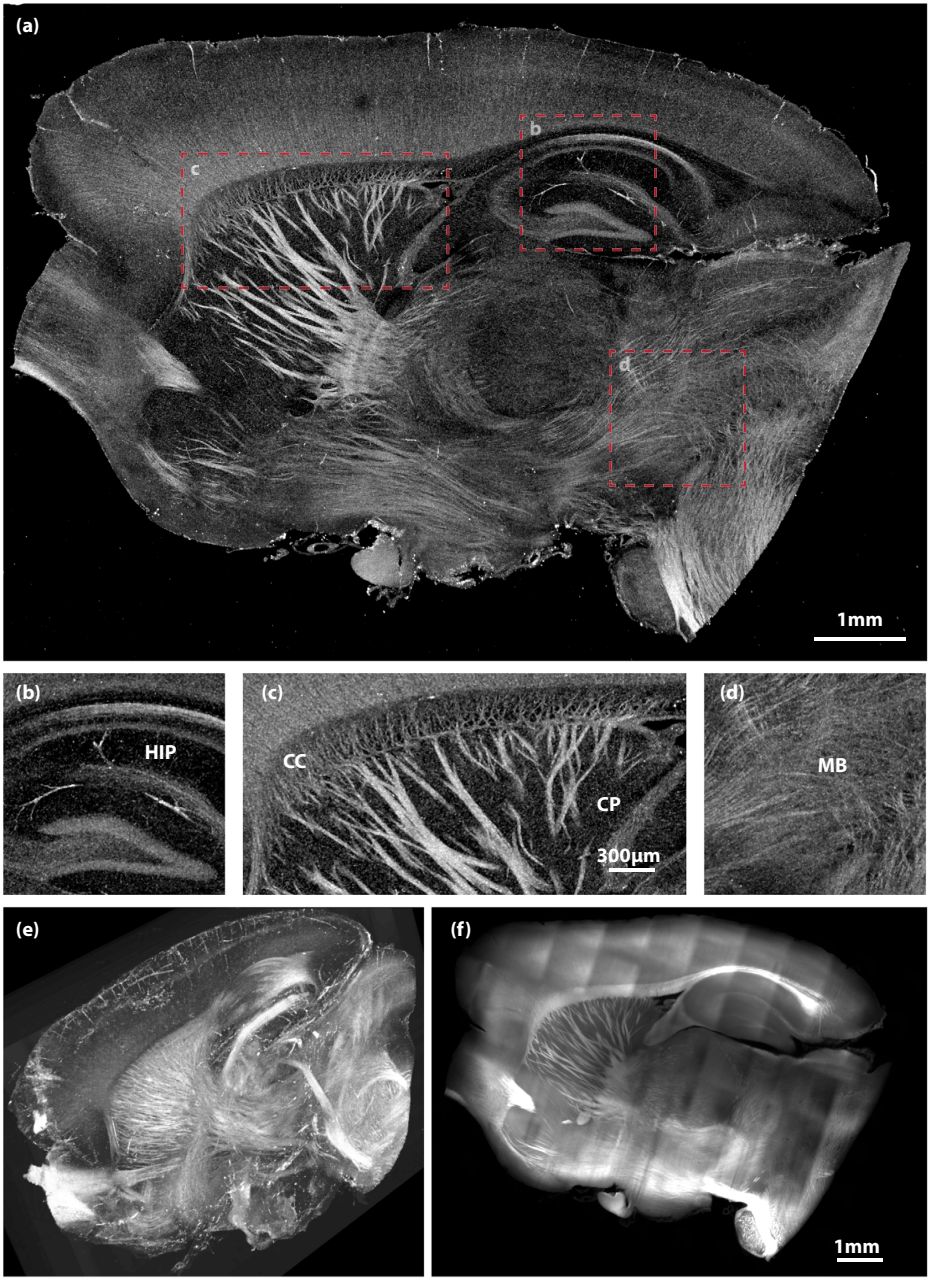
CAST imaging of an intact mouse brain hemisphere. (**a**) A sagittal view of the hemisphere. (**b**–**d**) Magnified images of the boxed areas in (**a**). (**b**) Hippocampus (HIP); (**c**) Corpus callosum (CC) and caudoputamen (CP); (**d**) Midbrain (MB). (**e**) A three-dimensional rendering of the CAST volumetric image stack. (See Supplementary Movie 1 for a movie.) (**f**) The corresponding sagittal image from a light-sheet microscope on the same hemisphere stained by DiD. (**b**–**d**) share the same scale bar in (**c**).

After validating this methodology, we performed experiments to show the three-dimensional, deep imaging capability of CAST. Both a 1 *mm* thick coronal section and a right hemisphere of mouse brain were cleared and then scanned. Figure 3 displays imaging results of the coronal section while Fig. 4 shows different views of the hemisphere. To better visualize the three-dimensional image stacks, volumetric data with a height of 36 *μm* were projected to form the two-dimensional *enface* images. Various regions with high content of white matter tracts were clearly visualized, such as corpus callosum (CC) (Figs 3(d) and 4(c)), caudoputamen (CP) (Figs 3(b) and 4(c)), thalamus (TH) (Fig. 3(e)), and globus pallidus (GP) (Fig. 3(c)). One can see the thalamocortical radiations, the fibers connecting between the thalamus and the cerebral cortex, emerge from thalamic basal ganglion, extend via the internal capsule (IC) into CP, and eventually project to the cortical layers. The vascular network in the cortex was also highlighted simultaneously. While axonal bundles and vessels were particularly enhanced, some grey matter were also detected. For example, the layered structure of the hippocampus (HIP) (Fig. 4(b)) was disclosed together with some prominent vessels inside. Additionally, some neuronal fibers within the cortex (Fig. 3(d)) were also captured.

A three-dimensional rendering of the hemisphere is shown in Fig. 4(e) (See Supplementary Movie 1 for a movie). The overall dimension for this stack is 10 *mm* × 7.5 *mm* × 6.2 *mm*. Due to the limited depth of focus, an axial focus stepping (a total 13 steps with an interval of 350 *μm*), similar to the Fig. 1, was employed. A method was developed for CAST to fuse the stepped images, i.e. merging different visualized areas in Fig. 1(e–h). While the focus stepping approach appears to work well for this application, the depth of focus limit can alternatively be extended by computational methods^34^. To better understand the structures observed in the three-dimensional CAST image, lipophilic dye DiD was applied to the sample to highlight the residual lipid on cell membranes and thus disclose the basic structure. The stained sample was subsequently imaged by a light-sheet microscope (Method). As rendered in Fig. 4(f), the structures in both images have good correspondence. To further validate that CAST provides an accurate representation of tissue structures throughout the entire volume, the images acquired by both methods are compared across the entire imaging volume. As shown in Supplementary Figure 1, structures disclosed by both modalities are close to identical. With CAST, visualizing both long range connections and local projections at the mesoscale is possible. Because the images were acquired on intact tissue in one volumetric data set, continuous fiber tracking would not require the complex spatial registration of multiple slices as is required with sectioning methods. The results presented in Figs 3 and 4 clearly demonstrated that this method, CAST, for the first time to our knowledge, enables deep imaging into intact tissues without any contrast reporter.

## Discussion

The resolution of our current system is not sufficient for identifying individual cells as the system was intentionally designed to balance speed and resolution. OFDI or optical coherence microscope (OCM) platforms with cellular resolution have already been demonstrated and are evolving into mature research tools^35^. Combined with the extended depth of focus, CAST may be capable of volumetric imaging with cellular resolution. Since it scans samples non-invasively, CAST is compatible with subsequent targeted, higher resolution investigation of biological structure with other modalities. Considering the significantly faster volume image acquisition, a possible role for CAST may be for rapid in-process monitoring of high-throughput studies and rapid pre-screening prior to applying higher resolution and exogenous-labeling methods.

By detecting light coherently, CAST is fundamentally distinct from other existing methods based on incoherent detection. This allows the measurement of not only the intensity but also subtle characteristics of light such as phase and polarization, opening the possibility of a wide spectrum of new contrast mechanisms in cleared tissue. At the same time, the speed of all fluorescence methods will be ultimately limited by the fluorescence throughput while the speed of CAST is theoretically limited by shot noise during detection because there is no similar ‘photo-bleaching’ effect on light scattering.

While individual neurons are the basic building blocks of the nervous system, brain function relies more on the interaction among the massive number of neurons. These connections exist across a broad range of scales. The scattering based imaging methodology described in this work offers a high-speed, label-free imaging alternative with a mesoscale resolution that potentially links connectomes across broad spatial scales.

## Methods

### Optical Frequency Domain Imaging (OFDI) System

OFDI is particularly advantageous for volumetric imaging in that it offers a unique depth scan (A-scan) along the probing beam. Together with transverse sampling, three-dimensional image stacks can be obtained.

A customized OFDI imaging system was developed for this work. The system was based on an optical interferometer, where light from a wavelength swept laser was first branched into a sample arm and a reference arm, and then the returned light from tissue sample and a reference mirror was combined, interfered, and detected. The homemade wavelength swept laser utilized a semiconductor optical amplifier (*Covega Corp., BOA-4379*) as the gain medium and a polygon scanner (*Lincoln Laser Co., SA34/DT-72-250-025-AA/#01B*) as the tunable filter to rapidly tune the wavelength at a rate of 54 *kHz*. This source had a center wavelength of 1300 *nm* and a sweeping range of 110 *nm*, resulting an axial resolution of 9 *μm* in air. It eventually delivered 10 *mW* optical power at sample. By employing an acousto-optic frequency shifter (*Brimrose Corp., AMF-50-1300*), the imaging depth of the system was extended to 6 *mm*.

The resulting interference fringes, as a function of wavelength, were detected by a polarization diversity demodulator to avoid polarization artifacts. A pair of balanced detectors (*Thorlabs Inc., PDB110C*) were used to measure the interference fringes from two orthogonal polarization channels. The interference signals were captured by a two-channel 14 *bit PCIe* digitizer board (*DynamicSignals LLC., PX14400A*) at 85 *MHz*, resulting in 1024 pixels per axial scan and an axial sampling pitch of 5.86 *μm*. A customized application based on *C*++ was developed to continuously stream the sampled data to a RAID hard-drive array. The application also processed and rendered the OFDI images at approximately 40% of the acquisition rate, allowing for real-time monitoring on image acquisition sessions.

The probing beam from a single mode fiber (*Corning Inc., SMF-28*) at the sample arm was first expanded by a fiber collimator (*Schäfter Kirchhoff GmbH, 60FC-4-M20-08*) to a diameter about 4 *mm* and then focused by a 18 *mm* scan lens (*Thorlabs Inc., LSM02*), resulting a working distance of 7.5 *mm* and a lateral resolution of 10 *μm*. Transverse sampling was achieved by two orthogonal scan axes (*X, Y*). The fast axis, *X*, was implemented by scanning a galvo mirror (*Cambridge Technology Inc., D05822*) at the back focal point of the lens. In this work, a 7.5 *mm* transverse field along *X* was scanned at a sampling pitch of 3.67 *μm*. This resulted in 26.4 frames per second with each frame comprised 2048 axial scan. A motorized translation stage (*Newport Corp., VP-25XA*) was used to translate samples along the slow axis, *Y*. A translation speed of 96 *μm*/*sec* was chosen to maintain an isotropic sampling. The scanned field along this direction was determined interactively based on tissue dimension. The axial scan and the transverse scans (*X, Y*) were all synchronized with the acquisition by the custom application.

The volumetric imaging of brain hemisphere in Fig. 4 employed 13 transverse scans stepped along axial direction, covering a depth of 6.2 *mm*. The scanned field along the slow axis was determined to be 10 *mm*. Overall, the entire acquisition took about 22 minutes with each transverse scan taking about 1.5 minutes.

### Tissue Preparation

SWITCH^26^, a gel-embedding based clearing method close to CLARITY^22^, was particularly chosen as our protocol of tissue preparation. This choice was mainly due to two advantages of SWITCH. First, the extent of lipid removal can be well controlled during its detergent clearing step. Second, the gel matrix preserves most native bio-molecules thus allowing further immunostaining to validate the contrast obtained from OFDI.

Mouse brain tissues from young adult wild type (C57BL/6) mice aged 6–8 weeks, were first preserved via a cardiac perfusion. The procedure started with perfusing 20 *mL* of ice-cold PBS followed by 20 *mL* of the ice-cold perfusion solution (1 × PBS, 4% paraformaldehyde (PFA), and 1% glutaraldehyde (GA)). Upon the completion of the perfusion, brain tissues were removed from the skulls and incubated into 20 *mL* of perfusion solution with gentle shaking for 3 days at 4 °C. Then samples were washed twice in PBST with 6 *hrs* each at room temperature with gentle shaking. After the initial wash, samples were washed overnight in inactivation solution (1 × PBS, 4% (*w*/*v*) acetamide, and 4% (*w*/*v*) glycine) at 37 °C. After another two washes with 6 *hrs* each in the clearing solution (200 *mM* sodium dodecyl sulfate (SDS) and 20 *mM* sodium sulfite), inactivated samples were incubated in the clearing solution with 70 °C water bath for lipid removal. Various clearing time and temperature were applied to achieve different levels of optical transparency on samples with different sizes.

Cleared samples were labeled with DiD (*ThermoFisher, D7757*) and two stains, DyLight 488-conjugated tomato lectin (*Vector Laborotories, DL-1174*) for vessels, SMI-312 antibody (*BioLegend, 837904*) and Alexa Fluor 594-conjugated donkey anti-mouse IgG antibody (*Abcam, ab150112*) for neurofilaments. Samples were first incubated in label-OFF solution (1 × PBS, 0.5 *mM* SDS for antibodies or 10 *mM* SDS for DiD) to reach equilibrium before transferred to a fresh buffer of label-OFF solution with antibodies or other bio-markers added. The samples were incubated in the buffer at 37 °C with gentle shaking for 12 *hrs* to 7 days depending on their sizes. Finally, samples were incubated in PBST at 37 °C for 2 days.

Before imaging, samples were incubated in a refractive index matching solution (23.5% (*w*/*v*) n-methyl-d-glucamine, 29.4% (*w*/*v*) diatrizoic acid, and 32.4% (*w*/*v*) iodixanol) over-night at room temperature.

### Fluorescence Imaging of Stained Samples

A confocal fluorescence microscope (*Olympus, FluoView1200MPE*) was used in this study. The system was configured with a 10× objective (*Olympus, XLPLN10XSVMP* 8 *mm WD* 0.6 *NA*) and three channels at 488, 559, and 635 *nm* were employed. Transverse sampling pitch was 1.59 *μm* and axial pitch was 20 *μm*. The overall acquisition time for each 1 *mm* thick sample was about 2 *hrs*.

A DiD-stained mouse brain hemisphere was imaged by a custom-built light sheet microscope. The sample was illuminated with a sheet of light generated by scanning a focused beam from an objective (*Olympus, Macro 4X/0.28 NA*) with a galvo-scanner (*Cambridge Technology, 6215 H*) and then imaged with a second objective (*Olympus, XLPLN10XSVMP* 8 *mm WD* 0.6 *NA*). Fluorescence signals were recorded by a sCMOS camera (*Hamamatsu, Orca Flash4.0 V2*). Its laser unit covered a broad spectrum (*Omicron, SOLE-6*), matching various emission filters. The dual illumination arms on both sides of the sample illuminated the sample simultaneously. Rolling shutter mode of the camera was utilized for dynamic confocal detection. Images were acquired by synchronizing the focal plane of the detection objective and the position of the illumination light sheet. Multiple image stacks were stitched with Terastitcher (*Bria, BMC Bioinformatics 2012*) and visualized with Imaris (*Bitplane*). Transverse sampling pitch was 2.32 *μm* and axial pitch was 5 *μm*. The total acquisition time was about 1 *hrs*.

All experimental protocols were approved by the MIT Institutional Animal Care and Use Committee and Division of Comparative Medicine and were in accordance with guidelines from the National Institutes of Health, US.

## Additional Information

**How to cite this article:** Ren, J. *et al*. Label-free volumetric optical imaging of intact murine brains. *Sci. Rep.* **7**, 46306; doi: 10.1038/srep46306 (2017).

**Publisher's note:** Springer Nature remains neutral with regard to jurisdictional claims in published maps and institutional affiliations.

## Supplementary Material

Supplementary Information

Supplementary Video
