# Supplementary material for: Label-free volumetric optical imaging of intact murine brains
Source: Sci Rep. 2017 Apr 12;7:46306. doi: 10.1038/srep46306 (PMC5388920; doi:10.1038/srep46306)
Supplement: Supplementary Information [file srep46306-s1.pdf]

Supplementary Information for 'Label-free volumetric optical imaging of intact murine brains'

Jian Ren, Heejin Choi, Kwanghun Chung, and Brett E. Bouma

January 3, 2017

1 Supplementary Movie 1 A mouse brain hemisphere imaged by CAST

We present a video to better visualize the 3D image volume of a mouse brain hemisphere acquired by CAST. Beside the stereoscopic rending, it also includes the fly-through movie of *en face* images. The scale bar varies accordingly for different frames.

2 Supplementary Figure 1: Colocalization of CAST and light-sheet images

To validate the accuracy of CAST, we compare CAST and light-sheet images across the entire volume of a mouse brain hemisphere. The images collected by both methods at various depths ranging from $0.7\,mm$ down to $4.7\,mm$ deep into the sample are contrasted in Supplementary Figure 1. The structures depicted by both methods are generally very close to identical throughout the entire tissue volume. It is worth noting that there exist some subtle discrepancies between the images. Although both data sets were acquired from the same sample, CAST imaging was performed prior to the DiD staining and the tissue structures reshaped very slightly during the staining process.

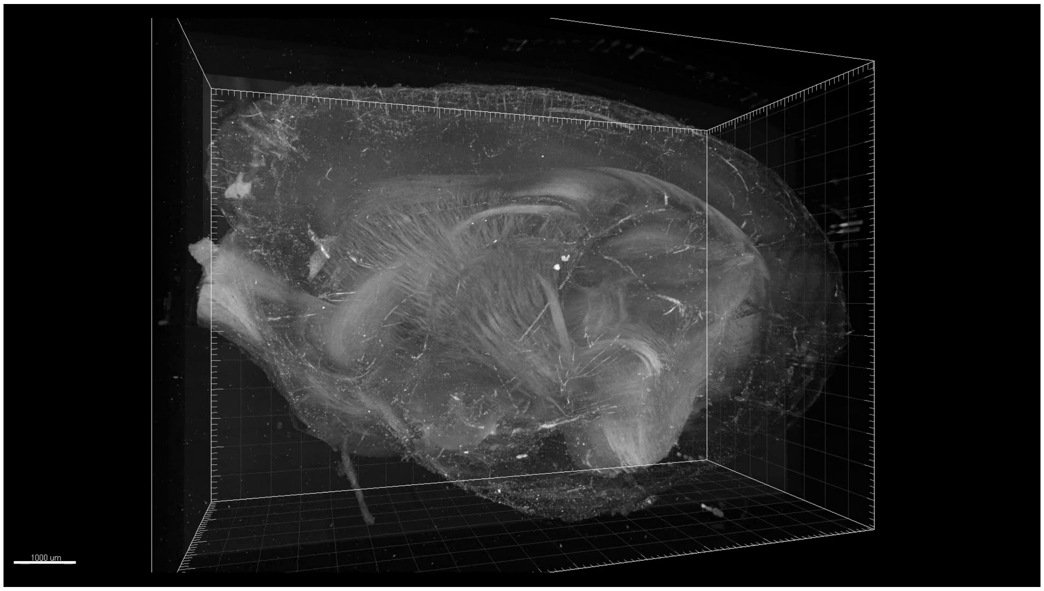

Supplementary Movie 1: A three-dimensional rendering of the CAST volumetric image of a mouse brain hemisphere. $46\,s$; $163.3\,MB$; 1920×1080; $H.264$

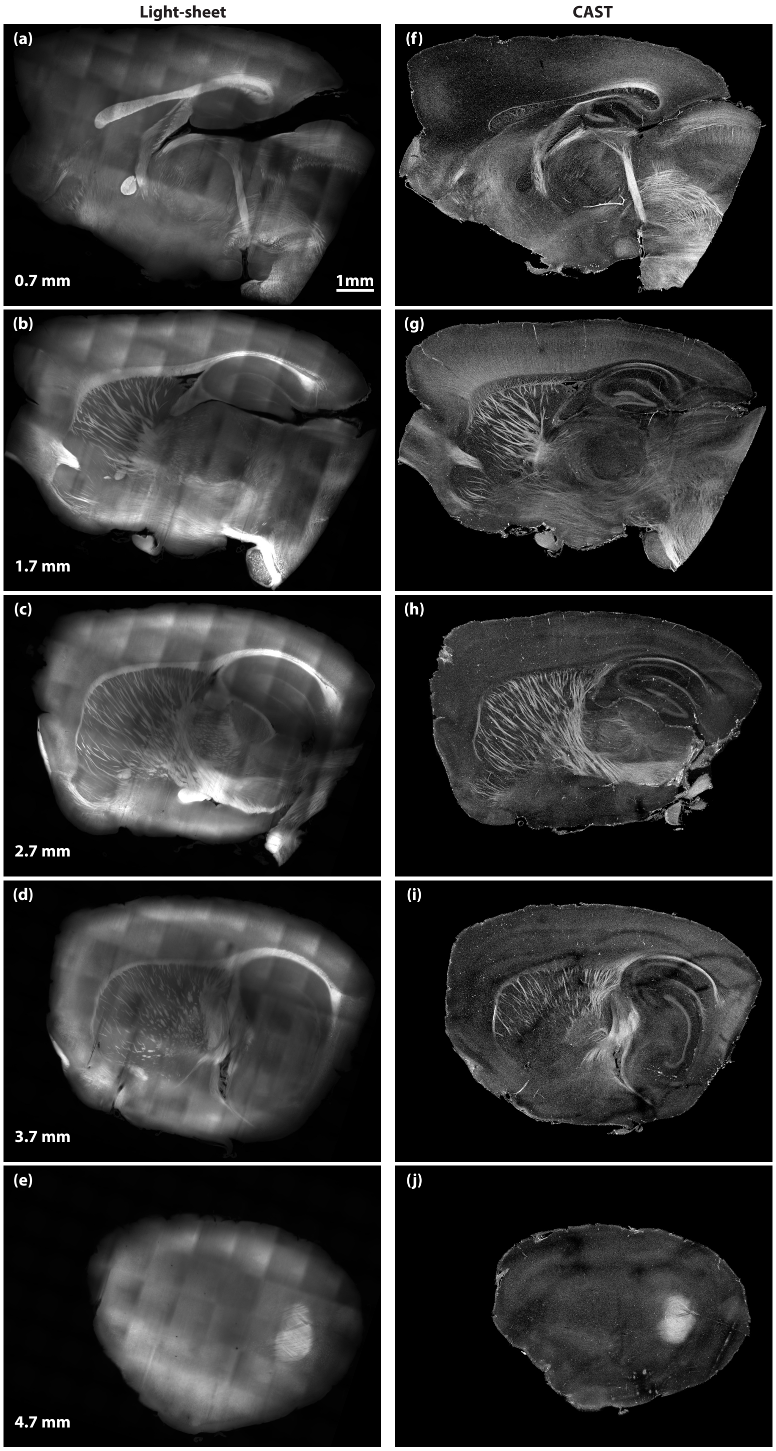

Supplementary Figure 1: Colocalization of CAST and light-sheet images. (a-e) The light-sheet sagittal images of a3 DiD-stained mouse brain hemisphere at depths of $0.7\,mm$, $1.7\,mm$, $2.7\,mm$, $3.7\,mm$, and $4.7\,mm$. (f-j) The CAST sagittal images of the same sample prior to DiD staining at the same depths as in (a-e). All images share the same scale bar in (a).
